# I Should but I Won’t: Why Young Children Endorse Norms of Fair Sharing but Do Not Follow Them

**DOI:** 10.1371/journal.pone.0059510

**Published:** 2013-03-20

**Authors:** Craig E. Smith, Peter R. Blake, Paul L. Harris

**Affiliations:** 1 Department of Psychology, University of Michigan, Ann Arbor, Michigan, United States of America; 2 Department of Psychology, Boston University, Boston, Massachusetts, United States of America; 3 Graduate School of Education, Harvard University, Cambridge, Massachusetts, United States of America; Boston College, United States of America

## Abstract

Young children endorse fairness norms related to sharing, but often act in contradiction to those norms when given a chance to share. This phenomenon has rarely been explored in the context of a single study. Using a novel approach, the research presented here offers clear evidence of this discrepancy and goes on to examine possible explanations for its diminution with age. In Study 1, 3–8-year-old children readily stated that they themselves should share equally, asserted that others should as well, and predicted that others had shared equally with them. Nevertheless, children failed to engage in equal sharing until ages 7–8. In Study 2, 7–8-year-olds correctly predicted that they would share equally, and 3–6-year-olds correctly predicted that they would favor themselves, ruling out a failure-of-willpower explanation for younger children's behavior. Similarly, a test of inhibitory control in Study 1 also failed to explain the shift with age toward adherence to the endorsed norm. The data suggest that, although 3-year-olds know the norm of equal sharing, the weight that children attach to this norm increases with age when sharing involves a cost to the self.

## Introduction

Recent work has demonstrated that young children have a sophisticated understanding of fairness. In the second year of life, children expect equal outcomes when two people receive resources from a third person and expect unequal outcomes following unequal effort [1], [2]. By 3 years of age, children apply principles of equality and merit in appropriate situations [3], [4]. However, despite this early understanding of fairness, young children have a self-interested bias when they themselves stand to benefit in resource allocation situations [5], [6], [7]. This bias creates a gap between the standards of fairness young children endorse for others and children's own sharing behavior. While various studies have focused on either children's thinking about sharing or their behavior when they are given a chance to share at a cost to the self (e.g., [8]), little attention has been paid to factors that can explain the gap between the two. The primary of goal the present research was to systematically investigate potential explanations for the gap. We used several variations of a simple allocation task to examine potential processes that cause and eventually close the gap between beliefs about fairness and actual behavior across development.

### Background

Early work on distributive justice found a judgment-behavior gap when comparing children’s responses to hypothetical resource allocation scenarios and actual allocation tasks [9], [10], [11]. Specifically, for hypothetical stories, children progressed through several levels of reasoning about fairness: from self-interested (ego-centric or protagonist-centric) reasoning, to a belief in strict equality, to merit- and need-based allocations. By contrast, when children were placed in situations similar to the hypothetical cases, they tended to favor themselves, thus falling short of the principles they used to describe fairness.

Subsequent research did not include direct comparisons of fairness judgments and actual behavior, but focused instead on factors that influence one or the other. Despite the lack of direct comparisons in the existing literature, the judgment-behavior gap remains apparent when comparing across studies. For example, in several studies young children make judgments about how to allocate resources between third party characters, and they tend to favor equal allocations [3], [8], [12], [13]. By contrast, when children’s natural behavior is observed, sharing with peers is infrequent in the preschool years [5], [6], [7]. Further, in standardized sharing paradigms, preschool-aged children endorse and create allocations of candy and stickers that favor themselves [14], [15], [16], [17], even when the peer who will receive the lesser amount is sitting in front of them [18]. One recent cross-cultural study included both an actual distribution task - between the child and an adult - and a third party distribution task involving two dolls [8]. While children in that study were not directly queried about norms, the results revealed a familiar pattern in most cultures, with children as young as 3 years splitting resources equally when the self was not a recipient, but hoarding resources when the self was a recipient and sharing was costly.

Thus, there is abundant evidence that children are aware of fairness standards at a young age, yet allocate resources unfairly when they stand to benefit. In the current study, we contribute to this line of research by exploring several possible explanations for the judgment-behavior gap. After first demonstrating that the judgment-behavior gap exists in a simple resource allocation task, we explored three possible explanations for the gap that have not yet been tested. First, young children may believe that a norm of fairness applies to others, but may not believe that the same norm applies to themselves Although this idea has been proposed as a possible explanation for the gap between judgment and behavior in development [9], [19], surprisingly it has never been tested for norms of fairness. Second, young children may believe that norms of fairness apply to everyone, but may also believe that others will not follow those norms in practice. This pessimistic view of others' sharing may serve as the basis, perhaps at an implicit level, for young children's hoarding behavior. This is plausible because young children do see their peers engage in hoarding behavior [5]. Third, consistent with research showing that young children often focus on desires when norms and desires are at odds [20], young children may feel obliged to follow a fairness norm, but fail to inhibit powerful desires that conflict with such fair sharing when it involves a cost to the self.

### Present Research

In the present research we used a sharing task based on the Dictator Game to make a quantitative comparison between children’s beliefs about sharing norms and their actual sharing. The Dictator Game (DG) is a standard economic game in which a participant can allocate a set of currency units between themselves and another person whom they will never meet [21]. The DG has been particularly useful for studying the cognitive mechanisms that influence giving behavior in adults [22], [23]. Adaptations of this task have been used with children between 3 and 9 years of age, using stickers as currency [14], [15], [24]. In order to maintain consistency with previous experiments, we also used stickers as a resource and told participants that the stickers belonged to them. Without granting children initial ownership of the resource, children might have believed that the stickers belonged to the experimenter, which would have altered the nature of the task. Establishing initial ownership of the resource is also consistent with traditional distributive justice tasks in which children earn the resource, thus establishing a claim on it.

In Study 1, children were assigned to one of two groups. In the Self-Share/Other-Norm group, children had the opportunity to actually share stickers with another child (Self-Share). These children were then asked how many stickers another child who did the same task *should* share (Other-Norm). Based on the existing literature, we predicted that younger children would share less than half of their stickers, whereas older children would share equally. By contrast, and based on existing research (e.g., [12]), we predicted that children of all ages would endorse the norm of equal sharing for the other child.

In the Self-Norm/Other-Share group, children were asked how much they themselves *should* share with the other child (Self-Norm), and were also asked to predict how much another child *had actually* shared (Other-Share). This design allowed us to evaluate two of the possible explanations described above for the anticipated gap between younger children’s norms and behavior. First, if young children apply a less costly standard of sharing to the self as compared to others, they will deny that they should share in the Self-Norm condition but claim that others should share in the Other-Norm condition. Second, if younger children share unequally because they suspect that other children will as well, they will not only deny that they should share equally in the Self-Norm condition, but also predict that others have behaved selfishly in the Other-Share condition.

In addition to these planned contrasts across the various versions of the allocation task, we also looked for evidence that individual differences in sharing behavior might be driven by differences in inhibitory control. We tested the third explanation proposed above, namely that children might struggle to enact a norm of equal sharing because of a difficulty inhibiting the desire to claim most or all of a resource for themselves. While inhibitory control has been investigated as a contributor to antisocial behavior in childhood (e.g., [25]), this is, to best of our knowledge, the first test of the potential association between inhibitory control and the prosocial behavior of sharing. Inhibitory control cannot fully explain age-related increases in fair sharing, because positive age-sharing associations are observed even when sharing is not costly for children [16], [26]. Nonetheless, we anticipated that increases in inhibitory control might partially mediate the age-sharing correlation. To assess this possibility, the inhibitory control of children in the Self-Share/Other-Norm group was assessed via two measures. The potential role of inhibitory control was further examined in Study 2 using a different approach, thus allowing evidence from multiple methods to converge on the question about its contributions.

Finally, children were asked to justify their responses. Of particular interest was whether older children would make more explicit references than younger children to the importance of fairness in explaining their behavior in the Self-Share task. Such a developmental trend would mirror age trends seen in studies of children's attributions of moral emotions (e.g., [27]). In such studies, younger children associate self-serving transgressions (e.g., getting a toy via stealing) with positive emotions, and explain these emotion attributions with references to satisfied desires. However, by about 8 years of age, children often expect negative emotions to result from self-serving transgressions, and explain such expectancies with references to fairness violations and concerns about harm. Paralleling such studies of moral emotion attribution, children in the present research were faced with a potential conflict between desire satisfaction (keeping more of the resource for the self), and adherence to a norm (sharing). Hence, we anticipated that references to desires might decline with age and references to fairness norms might increase. With this developmental prediction in mind, we studied three groups of children: 3–4-year-olds, 5–6-year-olds, and 7–8-year-olds. These age divisions are similar to those used in studies of moral emotion attributions (e.g., [28]), as well as those used in previous research on children's sharing [16].

## Study 1

### Method

#### Ethics statement

The Harvard University Committee on the Use of Human Subjects in Research (IRB Registration Number: 00000109) approved the ethics of this study. Informed consent, in written form, was obtained from the parents of all children who participated in this study. Children provided verbal assent that they wanted to take part in the research.

#### Participants

Children ranging from 3–8 years (*n* = 102; 47 boys; *M_age_* = 6.25, *SD* = 1.42) were recruited in the Living Laboratory, a space set aside for child development research at the Boston Museum of Science. Three age groups were tested: (1) 3–4-year-olds (*n* = 28; *M*_age_ = 4.47; *SD* = .50); (2) 5–6-year-olds (*n* = 41; *M*_age_ = 6.14; *SD* = .47); and (3) 7–8-year-olds (*n* = 33; *M*_age_ = 7.90; *SD* = .50). In line with museum guidelines, data were not collected on ethnicity and socioeconomic background. However, a recent survey found that families that participate in studies at this location are primarily white and are headed by parents with post-secondary degrees.

In both Study 1 and Study 2, the numbers of children in the three age groups were not balanced because participants were recruited in a museum setting as they approached the Living Laboratory Space. To allay potential concerns about this issue, initial analyses were run in each study with equal-sized age groups. In both Study 1 and 2, participants in the larger age groups were selected randomly to create age groups that were the same size as the smallest group (e.g., in Study 1, data from all 28 of the 3–4-year-olds were analyzed along with data from 28 randomly-selected 5–6-year-olds and 28 randomly-selected 7–8-year-olds). The results of these analyses mirrored the results that emerged from analyses of the full sample. Thus, the findings from the full sample are reported below.

#### Procedure

Parents completed a short questionnaire concerning their child’s birthdate, gender, and birth order. Children sat across from the experimenter at a small table, and parents remained nearby, in view of their children.

Children were assigned to one of two groups. In the Self-Share/Other-Norm group (*n* = 58; 11 3–4-year-olds; 26 5–6-year-olds; 21 7–8-year-olds), children could actually share with another child (Self-Share), and were also asked how much another child *should* have shared in the same situation (Other-Norm). In the Self-Norm/Other-Share group (*n* = 44; 17 3–4-year-olds; 15 5–6-year-olds; 12 7–8-year-olds), children were asked how much they themselves *should* share (Self-Norm), and to predict how much another child did *actually* share (Other-Share). The two groups did not differ in terms of age or gender distribution.

All children were asked to indicate their favorite color from a choice of 6, and received 4 smiley-face stickers of that color. Four stickers were used to ensure that the youngest children would be able to represent an even split of the resource without advanced counting. To increase the value of the resource, children were shown that the stickers were of the scratch-and-sniff variety, and were asked to smell them. (A pilot test with a separate group of 13 girls and 12 boys confirmed that children aged 3–8 value these scented stickers highly. The pilot children were asked to rate how much they liked the stickers on a 3-point scale: 1 = not much; 2 = a little; 3 = a lot. They were asked to be honest, and were assured that their rating would not impact whether they received some of the stickers. The three age groups did not differ in their responses: (a) 3–4-year-olds (*n* = 8; *M* = 3.00); (b) 5–6-year-olds (*n* = 10; *M* = 2.90); (c) 7–8-year-olds (*n* = 7; *M* = 2.86); *F*(2, 22) = .52, *p* = .60. There were no gender differences, χ^2^ = .003, *p* = .95.).

The procedures for the Self-Share/Other-Norm and the Self-Norm/Other-Share groups are described separately below. The procedure was very similar for both groups, aside from key differences in the instructions to the children. Children in the Self-Share/Other-Norm group also received two inhibitory control measures. Children in the Self-Norm/Other-Share group did not receive these measures because the hypothesis about inhibitory control focused on the relationship between actual sharing and inhibitory control.

After receiving 4 stickers from the experimenter, children in the Self-Share/Other-Norm group were told that the stickers belonged to them. They were told that they could keep all of the stickers, or share any number (1–4) with another boy or girl who would be coming to the museum later (recipient gender was matched to participant). (See Protocol S1, available online, for all scripts.) Participants were given no further information about the recipient, and made their sharing choices in view of the experimenter. The public nature of the task set the scene for subsequent interview questions and allowed a direct comparison to the other tasks in which children had to respond publicly. Children placed any stickers that they wanted to give to the other child in an envelope. Any stickers they kept for themselves remained in front of them on the table. Children were then asked a justification question: *Why did you decide to keep ___ and give away ___?* Their responses were recorded verbatim.

Next, children in the Self-Share/Other-Norm group received two measures of inhibitory control: the Day-Night Task [29] and the Bear-Dragon Task [30]. The Day-Night Task always came first. In the Day-Night Task, children saw two pictures on a laptop computer - a sun and a moon - and were instructed to say “night” in response to the sun and “day” in response to the moon. Children practiced with four pictures, and were given feedback. In the test session, children saw 8 of each picture, in a pre-determined order. Each picture was displayed for two seconds, and was followed by a white screen for one second. Participants were credited with a correct response if they said the incongruent word (e.g., “night” for sun) before the next picture appeared. Participants who said the congruent word first, or who responded after the next picture had appeared, received no credit. Checks on the recording of children's responses were done by having two observers record responses in real time for 25% of the participants; there was 100% agreement.

In the Bear-Dragon Task, the experimenter demonstrated the six actions of the game (touching one's chin, top of head, stomach, nose, ears, and shoulders), and children were asked to imitate the experimenter. Next, the experimenter introduced a small bear and a small dragon. Participants were instructed to do what the bear asked, and to do nothing in response to the dragon. Memory for the rules was checked, and four practice trials were run (2 bear, 2 dragon). Children passed the practice session if they inhibited motion on the dragon trials (or tried to and showed recognition that they had made a mistake), and if they moved freely on the bear trials. All children showed an understanding of the rules by these criteria. Six bear trials and six dragon trials were presented in a pre-determined order. In each trial, the experimenter raised either the bear or the dragon (using the same hand across all trials), leaving the other puppet on the table. Children of all ages found the bear trials easy. Only the dragon trials were used in the analyses presented below [31]. Children were scored as follows: 0 = full movement, 1 = partial movement with subsequent inhibition, 2 = no movement. Two observers recorded children's responses in real time for 25% of the participants; agreement was nearly perfect, kappa = .98.

Finally, children in the Self-Share/Other-Norm group were presented with an envelope, ostensibly from a child who previously took part in the study. They were told that the other child could have shared 0–4 stickers with them, and were asked what the other child *should* have done. At the end of the procedure, children were given extra stickers to take home if they had given away most or all of their stickers.

Note that children in the Self-Share/Other-Norm group were always asked about their own sharing before they were asked about what the other child should have done. We did not counterbalance these questions because we were concerned that asking about what others should do might influence children's own sharing behavior, and such an effect was not of interest in the present study. The same type of reasoning influenced our decision to always ask the self-norm question first in the Self-Norm/Other-Share group.

We now describe the procedure that was used for children who were assigned to the Self-Norm/Other-Share Group. Children in this group were given 4 stickers by the experimenter and were asked to imagine that they had a chance to share 0–4 of them with another child. They were then asked to say how many of the stickers they *should* share with another child if they had the opportunity to do so. Children in the Self-Norm/Other-Share Group were then given an envelope, ostensibly from a boy or girl who had earlier faced a real choice about sharing his or her stickers. Children were reminded that the other child could have shared 0–4 stickers, and were then asked to make a serious guess about how many stickers the other child had shared.

### Scoring

After making sharing choices, predicting others’ sharing, or talking about what should be shared, participants were asked to justify their responses. Thus, a total of four sets of justifications were obtained, two from each group. Justifications were coded using the following categories, established *a priori*: (1) Norm-based: explicit references to a standard of being fair, equal, kind, or to wanting to make others happy; e.g., *Then it will be equal* and *I just wanted to be nice*; (2) Desire-based: explicit references to satisfying one’s own desires or to putting the self ahead of others; e.g., *It’s my favorite color* and *I want to keep them all*; and (3) Uncodable: responses that did not fit into the other two categories; e.g., *I don’t know*. When justifying their responses to the Other-Share and Other-Norm tasks, some children referenced their own desires (e.g., *I want to have a lot of stickers*), whereas others referenced the other child's desires (e.g., *She wanted to keep some)*. To increase statistical power, both types of desire-related responses were merged into the Desire-based category. A second coder classified a subset of 114 justifications, and interrater reliability was good, κ = .81. Discrepancies were resolved through discussion.

Children's responses on each trial of the Day-Night Task were scored as correct or incorrect; total scores could range from 0–16. Children's responses on each dragon trial of the Bear-Dragon Task were scored as either 0, 1, or 2; total scores could range from 0–12.

### Results

Because some studies have found evidence of gender [32] and birth order/sibling [16] effects on sharing, we checked for such effects in a series of preliminary analyses. No such effects emerged. Thus, subsequent analyses did not include these variables.

#### Analyses of sharing data

**Figure 1:**
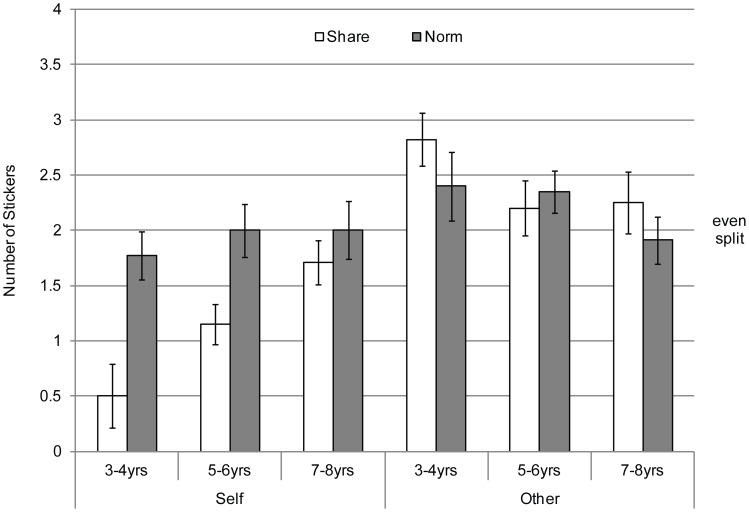
Children's mean responses to the four sharing-related tasks in Study 1 as a function of task type and age group.

**Figure 2:**
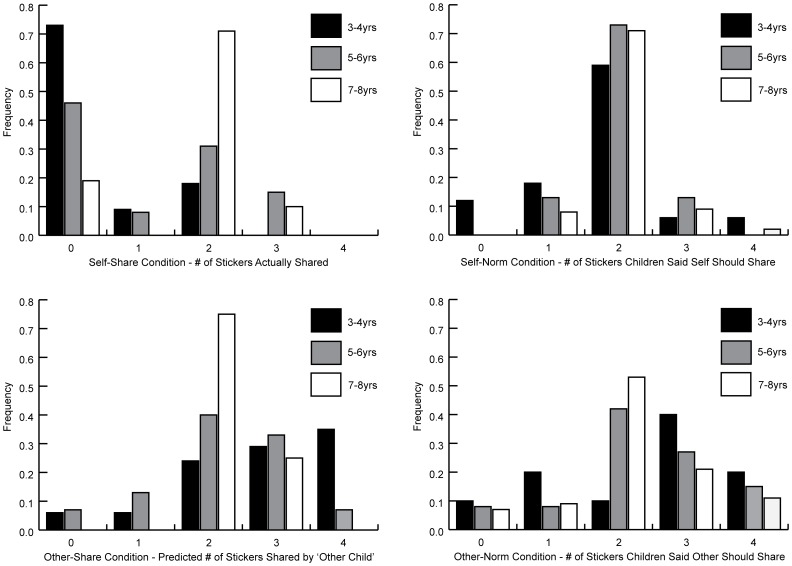
Frequencies of children's responses to the four sharing-related tasks in Study 1 as a function of task type and age group.

The Self-Share/Other-Norm group provided data on children's actual sharing behavior, and their beliefs about how much others should share. The Self-Norm/Other-Share group provided data on children's beliefs about how much they themselves should share, and how much they believed another child actually shared. Children's mean responses are displayed in Figure 1 as a function of age and response type (Share versus Norm) for Self and for Other. Figure 2 displays frequencies of children's responses across the four tasks.

In the Self-Share condition, one-sample *t*-tests (test value = 2 stickers, out of 4) confirmed what is apparent from inspection of Figure 1. Children of 3–4 years shared significantly less than half of the stickers (*M*_3–4 yrs = _.50; *p*<.001) as did 5–6-year-olds (*M*_5–6 yrs_ = 1.15; *p*<.001). By contrast, the sharing of 7–8-year-olds did not differ significantly from an equal split (*M*_7–8 yrs_ = 1.71, *p* = .16).

For the Other-Norm task, children of all ages judged that the other child *should* give them about half of the stickers; the means for the three age groups (*M*_3–4 yrs = _2.40; *M*_5–6 yrs_ = 2.35; *M*_7–8 yrs_ = 1.90) did not differ significantly from an even split (*ps >.35*). Likewise, for the Self-Norm task, children of all ages judged that they themselves should share about half the stickers (*M*_3–4 yrs = _1.76; *M*_5–6 yrs_ = 2.00; *M*_7–8 yrs_ = 2.00; *p*-values >.33). Finally, for the Other-Share task, children of all ages believed that the other child either shared significantly more than an equal split (*M*_3–4 yrs = _2.82, p<.01) or did not differ significantly from an equal split (*M*_5–6 yrs_ = 2.20; *M*_7–8 yrs_ = 2.25; *p*-values >.08).

In summary, irrespective of age, children judged that a norm of equal sharing applied to both themselves and others. Children also believed that other children adhered to this norm and had offered at least an equal share. Despite this widespread endorsement and expectation of equal sharing, younger children shared significantly less than an equal split when they actually had a chance to share. By 7–8 years of age, children adhered to the equal sharing norm. We examined this pattern of results more thoroughly in the analyses below.

First, to examine individual differences, children in the Self-Share/Other-Norm group were divided into two groups. One group was comprised of children who shared as much as or more than they said the other child should share. The other group was comprised of children who shared less than they said the other child should share. Table 1 displays the percentages of children who fell into each of these two groups as a function of age. Note that even among 7–8-year-olds almost one quarter of the children fell short of the norm they advocated. Nevertheless, a chi-square analysis confirmed what is evident in Table 1: there was a significant association between age and membership in the two groups, χ^2^(2, *N* = 57) = 7.84, *p* = .02. Follow-up analyses established that the 7–8-year-olds were more likely to enact their endorsed norm for others in the Self-Share task than were the 3–4-year-olds, χ^2^(1, *N* = 31) = 6.09, *p* = .01, and the 5–6-year-olds, χ^ 2^(1, *N* = 47) = 5.46, *p* = .02. The 3–4- and 5–6-year-olds did not differ, χ^ 2^(1, *N* = 36) = .46, *p* = .50.

**Table 1:** Percentages of children whose sharing lived up to or fell short of norms endorsed for others.^1^

	3–4 yrs	5–6 yrs	7–8 yrs
Actual Sharing<Norm for Others	70%	58%	24%
Actual Sharing ≥ Norm for Others	30%	42%	76%

1 In this analysis, children's own sharing was examined in relation to endorsed sharing norms for others because only children in the Self-Share/Other-Norm group made actual sharing choices. However, additional analyses confirmed that children's norms for the self and norms for others did not differ.

Next, for a direct comparison of children’s actual sharing and the sharing norms that children believed they themselves should follow, we conducted a 2-way ANOVA of Age Group (3–4, 5–6, & 7–8)×Response Type (Self-Share vs. Self-Norm). There was a main effect of Response Type, *F*(1, 96) = 18.68, *p*<.001, η_p_^2^ = .16. Across all ages, children in the Self-Share group shared fewer stickers (*M* = 1.22) than children in the Self-Norm group said they should share (*M* = 1.91). There was also a main effect of Age Group, *F*(2, 96) = 4.88, *p* = .01, η_p_^2^ = .09; both actual sharing and norms of sharing increased with age. The interaction effect fell short of significance, *F*(2, 96) = 2.28, *p* = .11. However, an analysis of simple effects was conducted, given the pattern revealed by the t-tests described above. As expected, actual sharing (Self-Share) was significantly lower than judgments about sharing norms (Self-Norm) for 3–4-year-olds (*F*(1, 96) = 13.85, *p*<.001) and 5–6-year-olds (*F*(1, 96) = 8.23, *p*<.01), but not for 7–8-year-olds (*F*(1, 96) = .75, *p* = .38).

Finally, for a direct comparison of children’s judgments about what other children should share and their predictions about what other children actually shared, we conducted a 2-way ANOVA of Age Group (3–4, 5–6, & 7–8)×Response Type (Other-Share vs. Other-Norm). There were no main effects of Age Group (*p* = .13) or Response Type (*p* = .32), nor was there a significant interaction (*p* = .44). In sum, at all ages children believed that other children *should* follow the norm of an equal split and *had* actually done so.

#### Inhibition tasks

In the next step of the analysis, we asked if children’s performance on the inhibition tasks was related to their sharing.

The Day-Night Task scores (*M* = 12.36, *SD* = 2.71) from the Self-Share/Other-Norm group were positively associated with age, *r*(56) = .53, *p*<.001. However, the scores from the dragon trials of the Bear-Dragon Task (*M* = 10.96, *SD* = 1.32) were not, *r*(55) = .10, *p* = .46. Children's performances on the two tasks were marginally associated, *r*(55) = .24, *p* = .07.

A positive and significant association between children's observed sharing behavior and inhibitory control emerged for the Day-Night Task, *r*(56) = .26, *p* = .05. No such relationship emerged between sharing behavior and performance on the Bear-Dragon task, *r*(55) = .03, *p* = .82. Given these trends, the focus of subsequent analyses was on the connection between Day-Night Task performance and sharing behavior.

Following Baron and Kenny [33], we tested the possibility that inhibitory control mediated the link between age and sharing. As an initial step, the independent variable, age, was again shown to predict the dependent variable, sharing behavior, *β* = .42, *t*(56) = 3.43, *p*<.01. Second, child age was again shown to predict Day-Night Task performance, *β* = .53, *t*(56) = 4.73, *p*<.001. Finally, sharing was regressed on both child age and inhibitory control. With both independent variables in the model, inhibitory control was no longer a significant predictor, *β* = .05, *t*(55) = .34, *p* = .73, whereas child age was, *β* = .39, *t*(55) = 2.69, *p*<.01. Thus, inhibitory control, as measured by the Day-Night Task, did not emerge as a mediator of the age-sharing association.

As a final step in exploring potential links between age, sharing, and inhibitory control, children in the Self-Share/Other-Norm group were divided into those that shared more than 0 stickers (*n* = 34) and those that shared nothing (*n* = 24). A 2-way ANOVA of Age Group (3–4, 5–6, & 7–8) × Sharing Group (something vs. nothing) was then used to predict scores on the Day-Night Task. Consistent with the correlation analysis reported above, there was a main effect of age, *F*(2, 52) = 4.25, *p* = .02, η_p_^2^ = .14; older children scored higher on the Day-Night Task compared to younger children. However, there was no main effect of sharing group, *F*(1, 52) = .41, *p* = .52, and the interaction term fell well short of significance, *p* = .96.

#### Children's justifications for their responses to the sharing tasks

Children were asked to justify their responses in all conditions. Justifications were coded using the three categories described earlier (Norm-based, Desire-based, and Uncodable); 77% of responses were codable. Table 2 presents the frequencies of children's Norm-based justifications.

**Table 2:** Frequencies of Norm-based justifications^2^ as a function of task type and age group.

Task Type	3–4 yrs	5–6 yrs	7–8 yrs
Self-Share (actual sharing)	20%	47%	81%
Self-Norm (sharing norms for self)	90%	100%	82%
Other-Share (guess about other's sharing)	50%	75%	100%
Other-Norm (sharing norms for other)	43%	81%	100%

2 Percentages reported here were computed using the pool of codable justifications provided by each age group in each task type. Thus, the percentages of Desire/Self-based justifications are differences between provided percentages and 100%.

First, justifications from children in the Self-Share group were compared to the justifications in the Self-Norm group at each age level. Fisher's Exact Tests were used for these analyses due to low expected counts in some cells. For the 3–4-year-olds, the percentage of Norm-based responses following actual sharing in the Self-Share task (20%) was significantly lower than the percentage of Norm-based responses following children’s statements of what they should share in the Self-Norm task (90%), p<.01. Similarly, the 5–6-year-olds also provided fewer Norm-based justifications in the Self-Share task (47%) compared to the Self-Norm task (100%), *p*<.01. However, the 7–8-year-olds provided Norm-based justifications with similar frequency following both the Self-Share (81%) and Self-Norm (82%) tasks, *p* = 1.00. In sum, only the oldest children frequently referred to ideas about fairness and kindness both after they actually shared and after they talked about what they should share.

Next, justifications by the Other-Share group were compared to justifications by the Other-Norm group at each age level. For the 3–4-year-olds, the percentage of Norm-based responses following the Other-Share task (50%) was not different from the percentage of Norm-based responses following the Other-Norm task (43%), *p* = 1.00. The 5–6-year-olds provided Norm-based justifications with similar frequency following the Other-Share (75%) and Other-Norm (81%) tasks, *p* = .69. Likewise, the 7–8-year-olds offered Norm-based justifications a similar percentage of the time following the Other-Share (100%) and Other-Norm (100%) tasks, *p* = 1.00. Thus, all three age groups provided norm-based responses equally often whether explaining their judgments about what the other child had shared or should share.

A trend in Table 2 warranted additional exploration. The 3–4-year-olds used Norm-based justifications in the Self-Norm task 90% of the time (considering only codable data). However, when responding to the Other-Norm task, the 3–4-year-olds, unlike the two older groups, used Desire-based justifications the majority of the time (57%). To interpret this pattern, young children’s answers in the Other-Norm task were examined in detail. Some talked about their own desires when justifying what the other child should share (e.g., *I want to have stickers*). Others talked about the desires of the other child, even as they stated that the other child should share (e.g., *She should share 3 because they were hers and she wanted to keep some*). Almost all 3–4-year-olds (90%) said that they themselves should follow the equal-sharing norm because it is fair, but some talked about how other children should follow the norm because it would satisfy the desires of one or both parties. This difference approached significance with the conservative, 2-tailed Fisher's Exact test, *p* = .10.

#### Mediation analyses for justifications

**Figure 3:**
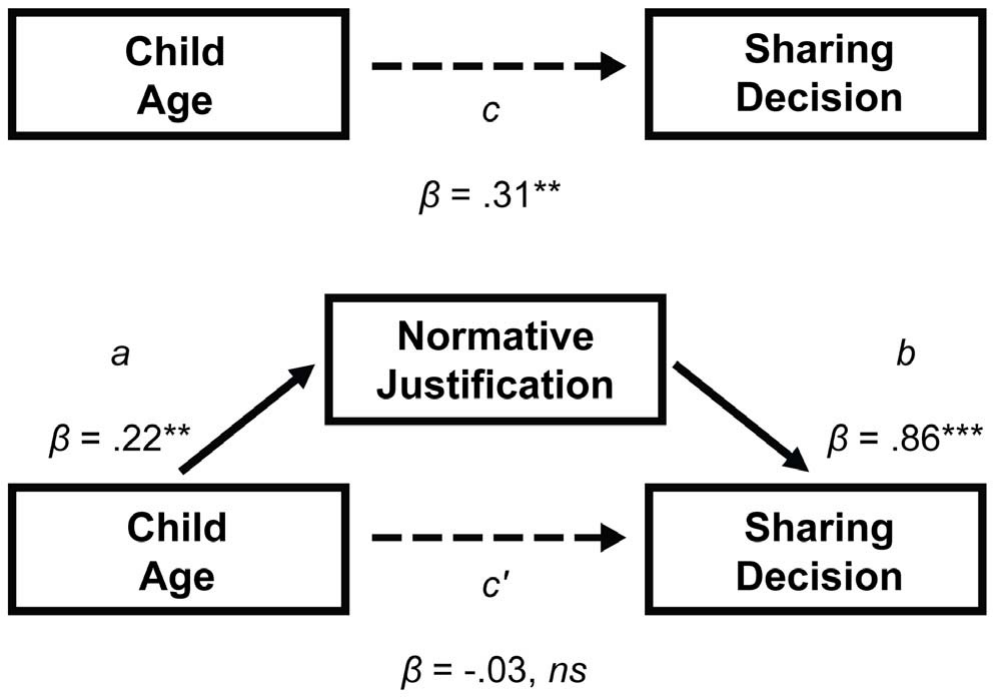
The indirect effect of child age on sharing via reasoning about norms vs. desires (^**^ *p*<.01, ^***^ *p*<.001). (Note: An estimated standardized coefficient for path *a* was computed using an equation supplied by Jason E. King, unpublished manuscript.).

A second mediation model was tested to explain the positive association between age and actual sharing in the Self-Share/Other-Norm group. We examined whether explicit references to fairness or kindness would mediate the positive association between age and observed sharing. To test this possibility, children's Desire-based justifications (*n* = 21; coded as 0) and Norm-based justifications (*n* = 24; coded as 1) were used as the mediating variable. Again, a series of regression models (see Figure 3) was calculated using equations designed for testing mediation models containing dichotomous mediators [34]. First, the independent variable, age, was again shown to predict the dependent variable, sharing behavior, *β* = .42, *t*(56) = 3.43, *p*<.01. Second, child age was again shown to predict justification type, *B* = .85, Wald = 8.02, *df* = 1, *p*<.01. Finally, sharing was regressed on both child age and justification type. With both independent variables in the model, child age was no longer a significant predictor, *β* = -.03, *t*(55) = -.46, *p* = .65, whereas justification type was, *β* = .96, *t*(55) = 17.41, *p*<.001. A Sobel test confirmed the indirect positive effect of child age on sharing via normative reasoning (test statistic = 2.85, *p*<.01). Therefore, strong support was found for the hypothesis that, as they get older, children's sharing increases because they increasingly think about how standards of fairness apply to their actual sharing behavior.

### Discussion

Study 1 confirms, in the context of a single study, that young children apply a norm of equal sharing both to themselves and to others before they actually follow that norm themselves. By about 8 years of age, children’s observed sharing aligned more closely with sharing norms, approximating an equal split.

The early gap between actual sharing and the sharing norm that young children endorse for others cannot be explained by two of the factors proposed earlier. First, young children do not fail to share equally because they deny that a norm of equal sharing applies to others *and* to the self. In the present study, even young children said that they themselves should offer an equal split and often provided justifications related to being fair or kind. Second, we found evidence that young children do not fail to share equally because they expect their peers to engage in unfair resource distribution. Instead, 3–4-year-old children optimistically predicted that other children would offer more than an equal split, while the older children believed that other children would follow the norm. Admittedly, it is difficult to rule out the possibility that younger children were prone to wishful thinking when predicting others’ sharing behavior, as they themselves were the recipients. Future investigations of this particular question would benefit from better probe questions (e.g., predictions about sharing between third parties). Finally, in what we believe to be the first test of the link between inhibitory control and sharing behavior in childhood, young children’s failure to share equally was not related to their performance on two measures of inhibitory control.

In contrast to these negative findings, children’s justifications were systematically related to their sharing behavior. Between 3 and 8 years, children exhibited increasingly explicit reasoning about how standards of fairness apply to their actual behavior. This age-related shift from a focus on desires to a focus on fairness accounted for the connection between age and sharing behavior in a mediation model. We acknowledge that the connections between age, justifications, and sharing behavior could be accounted for by alternative models, and this is discussed further in the General Discussion.

In sum, before age 8, young children assert that the norm of fairness applies not just to others but to the self and they reason explicitly about such norms when saying how they should share. However, they do not act on that norm when faced with a situation in which sharing a resource with others results in less for the self. Older children increasingly adhere to the sharing norm in costly sharing situations, and they increasingly invoke that norm when explaining their behavior.

In Study 2, we further explored the nature of the judgment-behavior gap seen in the younger children. Arguably, children are not knowingly hypocritical. They may plan to behave fairly, yet fall short of the sharing norm at the last moment when facing a sharing decision. Alternatively, young children might be aware, even before being faced with a sharing decision, that they will share less than they know they should. To examine these two possibilities, children were asked to predict how much they would offer when given an opportunity to share. This also allowed us to examine, from another angle, the hypothesis that inhibitory control impacts sharing. If young children do indeed plan to behave fairly, their failure to enact such plans could plausibly be attributed to problems with dominant-response inhibition – problems that might not have been adequately captured by the Day-Night and Bear-Dragon tasks. However, if the young children in Study 2 accurately predict that they will fall short of the sharing norm, this would further undermine the hypothesis that problems with inhibitory control lead to the type of self-serving behavior that we observed in young children in the Self-Share task.

## Study 2

In Study 2, we asked children to say not what they *should* offer, but to predict what they *would* offer when given an opportunity to share. Because young children in Study 2 were asked to think in the hypothetical about their sharing, it is important to note that prior research indicates that preschool-age children can make sense of simple, future-oriented hypothetical situations (e.g., [35], [36]). We anticipated two possible outcomes. First, given that children in Study 1 recognized that the norm for equal sharing applies to them, children might predict that they would follow that norm. That is, young children might have good intentions about equal sharing but fail to anticipate that their behavior will be governed by desires when faced with the actual costs of sharing. This line of explanation parallels research on moral hypocrisy in adults, showing that many adults want to be moral but succumb to temptation when the costs are high [37], [38]. Alternatively, children might accurately predict that they will favor themselves when sharing. In this case, we could conclude that young children know the norm of equal sharing but also know that they will not follow the norm.

### Method

#### Participants

Children ranging from 3–8 years (*n* = 59; 27 boys; *M_age_* = 5.82, *SD* = 1.20) were recruited in the Living Laboratory at the Boston Museum of Science. As in Study 1, children in Study 2 were divided into three age groups: (1) 3–4-year-olds (*n* = 18; *M*_age_ = 4.49; *SD* = .40); (2) 5–6-year-olds (*n* = 30; *M*_age_ = 5.90; *SD* = .44); and (3) 7–8-year-olds (*n* = 11; *M*_age_ = 7.76; *SD* = .50). All ethical guidelines noted in Study 1 were also followed in Study 2.

#### Procedure

Children again received 4 stickers of their favorite color. They were asked to imagine that they could share any number with another child, and were then asked to make a serious prediction about how many stickers they would share with another child if they had the chance (see Protocol S1, available online, for the full script).

### Results

Gender, birth order, and having a sibling did not relate to the outcomes of interest. Subsequent analyses did not include these variables.

#### Children's predicted sharing

The mean number of stickers the 3–4-year-old children anticipated sharing was.94 (*SD* = 1.16), significantly less than an equal split of 2 stickers, *t*(17) = -3.86, *p*<.001. The mean number of stickers the 5–6-year-olds predicted sharing was 1.37 (*SD* = .93) also significantly less than an equal split, *t*(29) = -3.74, *p*<.001. However, 7–8-year-olds predicted that they would share equally, *M* = 2.09, *SD* = .83, *t*(10) = .36, *p* = .72.

#### Comparison of predicted sharing with sharing norms

**Figure 4:**
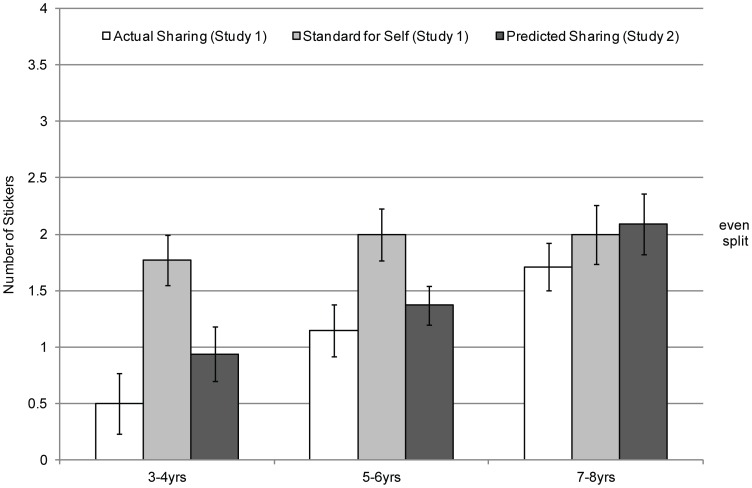
Children's mean responses to the actual sharing, self-norm, and self-prediction tasks across Studies 1 and 2 as a function of age group.

To assess whether children's sharing predictions in Study 2 were different from the self-norms endorsed by children in Study 1, a *t*-test was carried out within each age group. The data from these analyses are displayed in Figure 4.

Among the 3–4-year-olds, children's sharing predictions (*M* = .94) were significantly lower than the sharing norm they endorsed for the self in Study 1 (*M* = 1.76), *t*(33) = -2.26, *p* = .03. The 5–6-year-olds in Study 2 also predicted sharing fewer stickers (*M* = 1.37) than children of this age said they should share in Study 1 (*M* = 2.00), *t*(43) = -2.90, *p*<.01. By contrast, among the 7–8-year-olds, there was no difference between predicted sharing in Study 2 (*M* = 2.09) and endorsed sharing norms for the self in Study 1 (*M* = 2.00), *t*(21) = .75, *p* = .75.

#### Comparison of predicted sharing with actual sharing

To assess whether children's predictions about what they themselves would share (Study 2) were different from children's actual sharing (Study 1), another series of *t*-tests was carried out within each age group. The Self-Share data from Study 1 are also displayed in Figure 4.

In the 3–4-year-old group, children's actual sharing in Study 1 (*M* = .50) did not differ significantly from their predictions about what they would share in Study 2 (*M* = .94), *t*(26) = 1.06, *p* = .30. The 5–6-year-olds' actual sharing (*M* = 1.15) was also similar to their predicted sharing (*M* = 1.37), *t*(54) = .74, *p* = .46. Finally, among the 7–8-year-olds, actual sharing (*M* = 1.71) was also no different from predicted sharing (*M* = 2.09), *t*(30) = 1.15, *p* = .26.

### Discussion

Children were asked to predict what they would give to another child if they had the chance to share. Younger children correctly anticipated that they would not follow the norm of an equal split. Moreover, by ages 7–8, children correctly predicted that they would follow the norm of an even split. The results of Study 2 offer evidence that the judgment-behavior gap seen in Study 1 cannot be characterized by young children's failed intentions to live up to the equal-sharing norm. Young children reported no intention to live up to that norm.

## General Discussion

The current research extends traditional research on the development of fairness by assessing several possible explanations for the gap between children’s fairness judgments and their actual behavior. We first demonstrated that the judgment-behavior gap appears when using a modified version of a contemporary resource allocation task, the Dictator Game. We used this method to examine three possible explanations for the gap which had not yet been tested.

First, younger children may hold themselves to a lower standard of sharing than the one they endorse for their peers. However, younger children said that both they themselves and other children should share equally. Second, younger children may not expect other children to share equally. Yet, in fact, they expected other children to share at least half of the stickers in the same situation. Third, younger children may suffer from limited inhibitory control when faced with a conflict between the sharing norm and their impulse to take for themselves. Two pieces of evidence undermine this explanation. Children’s ability to inhibit a dominant response, as measured by the Day-Night Task, did improve with age, but this age-related improvement could not explain increases in sharing behavior with age. In addition, younger children correctly anticipated that they would share less than half. By implication, they did not suffer from a last-minute failure of will-power when faced with an actual decision. Instead, they were aware that they would share less than the norm even when they were asked to predict how many stickers they would share.

It could be argued that increases in fair sharing are due to children's developing concern for their moral reputations. On this account, children remain self-interested at all ages but, with age, they become more aware of the fact that other people’s opinions of them matter. The fact that the present studies were run in a public setting makes this line of reasoning initially plausible. Additionally, recent research has demonstrated that young children are more generous when their sharing behavior is visible to others [39], [40], and it is plausible that this sensitivity to the eyes of others becomes more pronounced with age. However, there are reasons to doubt a purely reputation-based account. Blake and Rand [15] found an age-related increase in equal sharing even when children made their sharing choices in private. Likewise, Benenson et al. [14] had the experimenter cover her eyes before children made sharing choices, yet an age-related increase in sharing still emerged. Thus, increased sharing with age occurs even in situations in which reputational concerns are diminished.

In addition to ruling out the explanations for the judgment-behavior gap discussed above, the present research supports an alternative account. Young children understand and accept that equal sharing is appropriate. However, they desire to keep the stickers for themselves. In the course of development, children increasingly give normative considerations weight in resolving such conflicts. On this hypothesis, the key developmental change is not an increasing ability to inhibit the impulse to satisfy their desires. Instead, it is an increasing acknowledgement of the force of normative considerations when faced with real situations that involve tensions between desires and norms. The pattern of justifications provides support for this explanation: younger children rarely referred to normative considerations. Instead, they often referred to their own desires when explaining both their predicted and their actual sharing behavior. By contrast, the oldest children mostly referred to normative considerations.

This analysis partly echoes traditional cognitive-structural theories of moral development in highlighting the moral reasoning that children deploy – both in making actual sharing decisions and in predicting their sharing decisions. Note, however, that the findings do not show that the specific content of children’s views on equal sharing change with age. When asked to say how they and other children should divide the resource and to predict how other children had divided the resource, children ranging from 3–9 years all focused on the norm of fairness. Thus, the age change was apparent only when children were invited to weigh such normative considerations against their own desires in the actual sharing task. By implication, it is the weight that children attach to those norms as a guide to their own behavior that changes with age rather than the content of the norms.

One plausible objection to this interpretation is that children’s justifications do not index the reflective process that actually preceded their sharing decisions. Instead, their justifications amount to accounts that children ‘invent’ retrospectively to explain what they have done [41]. We acknowledge that this objection cannot be decisively ruled out; additional studies involving pre-sharing priming are needed to better account for the role of norm-related cognitions. We note that a parallel developmental shift toward norm-based thinking can be observed in children's attributions of moral emotion (e.g., [27], [28]) even when they are not being asked to offer a retrospective explanation of their own behavior. When children are told about a protagonist who gets a toy he wants but harms someone else to get it, 4-year-olds often attribute positive emotions to the transgressor and focus on his satisfied desires. However, by age 8, children are much more likely to attribute negative emotions to the transgressor, and to focus on his normative transgression. We acknowledge that children in these “happy-victimizer” studies see the same stimuli at all ages, while the children in Study 1 were justifying sharing behaviors that differed across age groups. However, there is evidence that children's thinking across these very different paradigms is connected [24]. This may suggest that a robust developmental pattern exists in how children think about a wide range of situations involving tensions between satisfying desires and adhering to norms. The sharing paradigm used in the present study and the hypothetical scenarios used in research on the “happy victimizer” expectancy are two examples of these types of situations. Additional research in this area is needed to provide more clarity.

Two additional directions for future research deserve mention. First, recall that inhibitory control did not explain age-related shifts in sharing in Study 1. Still, it should be noted that keeping all of the stickers in the Study 1 sharing task did not require any action by participants; the stickers were already in their possession, and hoarding the resource was accomplished by simply staying still. Future studies should make use of tasks in which behaving fairly involves inhibiting a motor response (e.g., not taking more than one's fair share from a pile); such an approach may uncover associations with inhibitory control.

Second, the present research is unable to characterize the relationship between sharing norms and behaviors beyond age 8. Thus, it is impossible to claim, on the basis of the present research, that the norm-action gap closes and remains closed by age 8. The inclusion of a wider age range could plausibly reveal more than one age shift where the norm-action relationship in concerned. Indeed, recent research with participants ranging from 9 years of age up to adulthood suggests that adults may be less generous than teens when making monetary offers in a dictator game [42]. Further, studies on the topic of moral hypocrisy demonstrate that adults often fall short of behaviors that they view as generous or fair (e.g., [43], [44]). Clearly, the developmental complexity underlying fairness-related thought and action will be more fully uncovered in studies that include a wide age range. The use of tokens that are exchangeable for a wide range of valued items could make such a study possible, and could allow for the testing of other variables that may affect one age group differently than another (e.g., the salience of an observer during resource allocation).

### Conclusions

Studies 1 and 2 used an equivalent metric to compare: (1) how much of a resource children share with a peer, (2) what children say about how they and others should divide the resource, and (3) children’s predictions about how they and others will divide that resource. Children aged 7–8 years endorsed the norm of an equal split as fair, predicted that they and another child would behave in accordance with that norm, and actually did share equally with another child. Children aged 3–6 years also asserted that the resource should be divided equally, whether by themselves or by another child and they predicted that another child would adhere to that norm. Yet they predicted that they themselves would fall short of the norm, and actually did fall short. Age-related increases in inhibitory control failed to account for this closing of the judgment-behavior gap with increasing age. On the other hand, the extent to which children invoked fairness norms when reflecting on their actual sharing did explain the matching of behavior to standards that emerged among older children. The younger children focused on their own desires when explaining their predicted and actual sharing, whereas the older children talked spontaneously and explicitly about issues of fairness. The results provide some support for traditional accounts of moral development by showing that, in the course of development, children’s sharing is increasingly consistent with the norm of fairness that they endorse from an early age.

## Supporting Information

Protocol S1 **Full scripts.**
(DOCX)
