# Correction: I Should but I Won’t: Why Young Children Endorse Norms of Fair Sharing but Do Not Follow Them

**DOI:** 10.1371/annotation/4b9340db-455b-4e0d-86e5-b6783747111f

**Published:** 2013-08-21

**Authors:** Craig E. Smith, Peter R. Blake, Paul L. Harris

A funding organization and grant given to the first author were incorrectly omitted from the Funding Statement. The first sentence of the Funding statement should be: "Work on this research by CES was supported, in part, by Award Number T32HD007109 from the Eunice Kennedy Shriver National Institute of Child Health & Human Development." 

