# Supplementary material for: I Should but I Won’t: Why Young Children Endorse Norms of Fair Sharing but Do Not Follow Them
Source: PLoS One. 2013 Mar 20;8(3):e59510. doi: 10.1371/journal.pone.0059510 (PMC3603928; doi:10.1371/journal.pone.0059510)
Supplement: Protocol S1 — Full scripts. (DOCX) [file pone.0059510.s001.docx]

**Supporting Information for:**

***I should but I won't: Why young children endorse norms of fair sharing but do not follow them***

**PROTOCOL S1**

***Study 1 Self-Share Script.*** After receiving 4 stickers from the experimenter, children were told:

*Right now, all of these stickers are yours. If you want to, you can decide to share any number you want with another boy/girl who will come here later. If you decide to share some stickers with another boy/girl who will come here later, you can put the stickers you want to share in this envelope. Remember – all of these stickers are yours, and you can decide what you want to do. You could share 1 or 2 or 3 or 4, or you could keep them all for yourself. Okay – what do you want to do?*

***Study 1 Other-Norm Script.*** Children were presented with an envelope and were told that it was from a boy or girl who previously took part in the study. They then heard to following:

*This envelope is from a child who did this project with me before you came. He (or she) started with four stickers, just like you. He had to decide how many he wanted to share with you. He could have shared 1 or 2 or 3 or 4, or he could have kept them all for himself. Before we open this envelope to see what he did, I have a question for you. What do you think he should have done? Do you think he should have shared 1 or 2 or 3 or 4, or should he have kept them all for himself?*

***Study 1 Self-Norm Script.*** After children received 4 stickers from the experimenter, the experimenter asked them to say how many stickers they *should* share with another child using the following script:

*Right now, all of these stickers are yours, and you can take them all home when we’re done.  But, I want to ask you to imagine something.   Let’s imagine that you could share any number of these stickers with another boy/girl who will come here later.  Let’s imagine that if you decided to share some stickers with another boy/girl who will come here later, you could put the stickers you wanted to share in this envelope.  Remember – you really get to keep all of these stickers.  But what do you think you should do if you were really going to share them?  Do you think that you should share 1 or 2 or 3 or 4, or should you keep them all for yourself?*

***Study 1 Other-Share Script.*** Children given an envelope that was ostensibly from a child who had taken part in the study before they had. They were then asked to make a serious prediction about how much the other child had chosen to share using the following script:

*This envelope is from a child who did this project with me before you came. He (or she) started with four stickers, just like you. And, he really did have the chance to put stickers in here; he wasn’t just imagining that he would be sharing. You get to have whatever he put in there. Before we open this envelope to see what the other child did, I have a question for you. The other child had four stickers, and he could have kept them all or shared 1 or 2 or 3 or 4 with you. What do you think he decided to do?*

***Study 2 Self-Prediction Script:*** Children in Study 2 were given 4 stickers, and were asked to imagine that they had the chance to share 0-4 stickers with another child. They were then asked to make a serious prediction about what they would share using the following script:

*Right now, all of these stickers are yours, and you can take them all home when we’re done. But, I want to ask you to imagine something. Let’s imagine that you could share any number of these stickers with another boy/girl who will come here later. Let’s imagine that if you decided to share some stickers with another boy/girl who will come here later, you could put the stickers you wanted to share in this envelope. Remember – you really get to keep all of these stickers. But what do you think you would do if you could share them? Do you think that you would share 1 or 2 or 3 or 4, or would you keep them all for yourself?*
